# Retraction: Human Chorionic Gonadotropin β Induces Migration and Invasion via Activating ERK1/2 and MMP-2 in Human Prostate Cancer DU145 Cells

**DOI:** 10.1371/journal.pone.0314356

**Published:** 2024-11-19

**Authors:** The PLOS ONE Editors

After this article [1] was published, concerns were raised regarding Fig 6.

Specifically:

- In Fig 6A, the bottom region of the DV/- panel appears similar to the top region of the DH/+ panel.
- In Fig 6A, the bottom region of the DH/+ panel appears similar to the top region of the DH/- panel in Fig 6B.

The corresponding author acknowledged the similarity of the panels in Fig 6 and provided a corrected figure and the original microscopy images for the experiment.

Upon editorial review of the original microscopy images, further similarities were found between panels representing different experimental conditions.

In light of the above concerns that question the reliability of the underlying data and therefore the reported results and conclusions, the *PLOS ONE* Editors retract this article.

WW did not agree with the retraction and stands by the article’s findings. ZL, CL, LD and YZ either did not respond directly or could not be reached.

## References

[1] Li Z, Li C, Du L, Zhou Y, Wu W (2013) Human Chorionic Gonadotropin β Induces Migration and Invasion via Activating ERK1/2 and MMP-2 in Human Prostate Cancer DU145 Cells. PLoS ONE 8(2): e54592. 10.1371/journal.pone.0054592 23424616 PMC3570544

